# Supplementary material for: Organic cation transporter 2 contributes to SSRI antidepressant efficacy by controlling tryptophan availability in the brain
Source: Transl Psychiatry. 2023 Sep 29;13:302. doi: 10.1038/s41398-023-02596-y (PMC10542329; doi:10.1038/s41398-023-02596-y)
Supplement: Supplementary file 1 — Supplementary information [file 41398_2023_2596_MOESM1_ESM.docx]

**SUPPLEMENTARY INFORMATION**

**SUPPLEMENTARY MATERIAL AND METHODS**

**Behavioral tests**

The coat state was assessed weekly as a measure of motivation toward self-care. It was evaluated as the sum of the score of different parts of the body, ranging between 0 for a well-groomed coat and 1 for an unkempt coat for head, neck, dorsal/ventral coat, tail, and forepaws/hind paws. For the sucrose preference test, singled-house mice were first habituated for 48 h to drink water from two bottles. On the following 3 days, the mice could choose between a water bottle and a 1% (wt/vol) sucrose solution bottle, with the position switched daily. Sucrose solution intake for 24 h was measured during the last 2 days and expressed as a percentage of the total amount of liquid ingested. The social interaction test was performed in a white open-field (42 x 42 cm) containing an empty wire mesh cage (10 x 6.5 cm) located at an extremity of the field in a low luminosity environment (25 lux). Individual mice were allowed to explore the open-field for two consecutive sessions of 2.5 min. During the second session, an unfamiliar mouse was introduced into the wire mesh cage. Between the two sessions, the test mouse was placed back into its home cage for approximately one minute. The time spent by the test mouse in the interaction zone, defined as an 8-cm-wide region surrounding the mesh cage, was measured in both sessions by video tracking (Viewpoint). For the object location test, the mice were habituated during two successive days to an open-field containing an intra-field cue (one wall covered with black and white stripes). Each mouse was allowed to freely explore the open-field for a 30-min period on day 1 and for two 10-min sessions separated by 5 h on day 2. On the third day, the test mouse was allowed to explore for 5 min two identical objects (5 x 2.5 cm) positioned in two adjacent corners of the open-field (acquisition phase) then returned to its home cage for 1 h. For the sample phase trial, one of the two objects was displaced to the opposite corner of the open-field. The time spent exploring both objects was recorded over a 5-min session by video tracking. The elevated O-maze consisted of an annular runway positioned 40 cm above the floor and divided into two opposing 90° closed sectors and two 90° open sectors. Mice were individually placed in the closed sector and their behavior recorded over a 5-min period. The time spent in each sector and the number of sector entries (a sector entry was defined as all four paws being placed in a sector) were determined by video tracking (Viewpoint, Lyon, France).

**Western blotting**

Samples were homogenized by sonication in 2 vol of ice-cold phosphate-buffered saline containing 1% Triton X-100, protease inhibitors (Complete Protease Inhibitor Cocktail, Roche Diagnostics, Meylan, France) and phosphatase inhibitors (Phosphatase Inhibitor Cocktail 3; Sigma-Aldrich, Darmstadt, Germany). Protein concentrations were determined by Bradford’s method. Protein samples (15 μg) suspended in NuPage LDS sample buffer (Invitrogen, Carlsbad, CA, USA) were separated by Bis-Tris sodium dodecyl sulfate polyacrylamide gel electrophoresis (10% gels) and transferred onto nitrocellulose membranes (Invitrogen). Transfer efficacy was controlled by Ponceau S staining. Unspecific binding sites were blocked with Tris-buffered saline containing 0.1% Tween-20 and 5% nonfat milk and membranes were immunoprobed with antibodies against Erk1/2 (1/1500, Cat# sc-135900, RRID:AB_2141283) from Santa Cruz Biotechnology (CA, USA), phosphorylated extracellular-signal regulated kinase1/2 (pErk1/2, 1/1000, Cat# 9101, RRID:AB_331646), p-P70S6 (1/500, Cat# 9206, RRID:AB_331768), Akt (1/2000, Cat# 2920, RRID:AB_1147620), pThr308 (1/200, # 4056, RRID:AB_331163) or Ser473 Akt (1/200, Cat# 9271, RRID:AB_329826) and phosphorylated GSK3β (p GSK3β, 1/1000, Cat# 9336, RRID:AB_331406) from Cell Signaling (Danvers, MA, USA), glycogen synthase kinase-3β (GSK3β, 1/2000, Cat# 4414, RRID:AB_259907) or β-actin (1/2500; Cat# 4700, RRID:AB_476730) from Sigma-Aldrich. Membranes were incubated with infrared-labeled secondary antibodies (IRDye 700DX RRID:AB_220144 and IRDye 800CW, RRID:AB_220150; 1/5000) from Rockland (Gilbertsvillle, PA, USA). Immunoblotting was quantified with an Odyssey Infrared Imaging System and Application Software version 3.0 (LI-COR Biosciences, Lincoln, NE, USA). The experiments were replicated twice.

**SUPPLEMENTARY RESULTS**

**Supplementary table S1 Statistical analysis of behavior and coat state (Figure 1)**

Two-way ANOVA (n = 10-14) followed by Tukey’s post hoc test for behavior and Dunnett’s for coat state. Main effects of tryptophan supplementation, genotype and/or genotype*tryptophan supplementation interaction

| **Groups** | **Significance** |
| --- | --- |
| Sucrose preference  BASAL: *OCT2^+/+^* n = 14; *OCT2^-/-^* n = 14  CORT: *OCT2^+/+^* n = 14; *OCT2^-/-^* n = 14  CORT/FLUOX: *OCT2^+/+^* n = 11; *OCT2^-/-^* n = 13 | Treatment F2, 74 = 22.43; P < 0.0001  Genotype F1, 74 = 4.999; P = 0.0284  Interaction F2, 74 = 8.820; P = 0.0004 |
| Social interaction  BASAL: *OCT2^+/+^* n = 9; *OCT2^-/-^* n = 10  CORT: *OCT2^+/+^* n = 11; *OCT2^-/-^* n = 12  CORT/FLUOX: *OCT2^+/+^* n = 11; *OCT2^-/-^* n = 13 | \| Treatment F2, 60 = 9.377; P < 0.0003  Genotype F1, 60 = 1.204; P = 0.2769  Interaction F2, 60 = 2.642; P = 0.0795 \|  \| \| --- \| --- \| |
| Object location  BASAL: *OCT2^+/+^* n = 10; *OCT2^-/-^* n = 10  CORT: *OCT2^+/+^* n = 10; *OCT2^-/-^* n = 10  CORT/FLUOX: *OCT2^+/+^* n = 11; *OCT2^-/-^* n = 11 | \| Treatment F2, 56 = 3.863; P = 0.0268  Genotype F1, 56 = 11.09; P = 0.0015  Interaction F2, 56= 2.886; P = 0.0642 \|  \| \| --- \| --- \| |
| Elevated O-maze  BASAL: *OCT2^+/+^* n = 11; *OCT2^-/-^* n = 11  CORT: *OCT2^+/+^* n = 11; *OCT2^-/-^* n = 11  CORT/FLUOX: *OCT2^+/+^* n = 11; *OCT2^-/-^* n = 12 | \| Treatment F2, 61 = 43.30; P < 0.0001  Genotype F1, 61 = 0.3589; P=0.5513  Interaction F2, 61 = 5.737; P = 0.0052 \|  \| \| --- \| --- \| |
| Coat state  CORT/FLUOX: *OCT2^+/+^* n = 14; *OCT2^-/-^* n = 14 | \| Treatment F10, 280= 60.28; P < 0.0001  Genotype F1, 288 = 8.547; P < 0.0037  Interaction F10, 280= 1.170; P=0.3110 \|  \| \| --- \| --- \| |

**Supplementary table S2 Statistical analysis of HPLC data at basal state in brain and plasma (Figure 2A)**

Unpaired two-tailed student’s *t* test

| **Groups** | **Significance** |
| --- | --- |
| Striatum, Tryptophan and kynurenine  *OCT2^+/+^* n = 6; *OCT2^-/-^* n = 6 | Tryptophan  *OCT2^+/+^* vs *OCT2^-/-^*, p =<0.0001  Kynurenine *OCT2^+/+^* vs *OCT2^-/-^*, p = 0.0340 |
| Cortex, Tryptophan and kynurenine  *OCT2^+/+^* n = 6; *OCT2^-/-^* n = 6 | Tryptophan  *OCT2^+/+^* vs *OCT2^-/-^*, p = 0.2202  Kynurenine *OCT2^+/+^* vs *OCT2^-/-^*, p = 0.0174 |
| Hippocampus, Tryptophan and kynurenine  *OCT2^+/+^* n = 6; *OCT2^-/-^* n = 6 | Tryptophan *OCT2^+/+^* vs *OCT2^-/-^*, p = 0.0069  Kynurenine *OCT2^+/+^* vs *OCT2^-/-^*, p = 0.0780 |
| Cerebellum, Tryptophan and kynurenine  *OCT2^+/+^* n = 6; *OCT2^-/-^* n = 6 | Tryptophan *OCT2^+/+^* vs *OCT2^-/-^*, p = 0.0074  Kynurenine *OCT2^+/+^* vs *OCT2^-/-^*, p = 0.0055 |
| Plasma, Tryptophan and kynurenine  *OCT2^+/+^* n = 6; *OCT2^-/-^* n = 6 | Tryptophan  *OCT2^+/+^* vs *OCT2^-/-^*, p = 0.1091  Kynurenine *OCT2^+/+^* vs *OCT2^-/-^*, p = 0.0362 |

**Supplementary tabl~~e~~ S3 Statistical analysis of behavior and coat state after tryptophan supplementation (Figure 3A-D)**

Two-way ANOVA (n = 5-8) followed by Tukey’s post hoc test for behavior and Dunnett’s for coat state. Main effects of treatment, genotype and/or genotype*treatment interaction

| **Groups** | **Significance** |
| --- | --- |
| Sucrose preference  BASAL: *OCT2^+/+^* n= 14; *OCT2^-/-^* n= 12  CORT: *OCT2^+/+^* n= 14; *OCT2^-/-^* n= 14  CORT/FLUOX: *OCT2^+/+^* n= 6; *OCT2^-/-^* n= 6  CORT/FLUOX/TRP: *OCT2^+/+^* n= 8; *OCT2^-/-^* n= 4 | Treatment F3, 72= 16.54; P < 0.0001  Genotype F1, 72= 2.297; P = 0.1340  Interaction F3, 72= 3.142; P = 0.0304  * Two-way ANOVA on CORT, CORT/FLUOX and CORT/FLUOX/TRP groups |
| Social interaction  BASAL: *OCT2^+/+^* n=6; *OCT2^-/-^* n= 5  CORT: *OCT2^+/+^* n= 14; *OCT2^-/-^* n= 14  CORT/FLUOX: *OCT2^+/+^* n= 6; *OCT2^-/-^* n= 6  CORT/FLUOX/TRP: *OCT2^+/+^* n= 8; *OCT2^-/-^* n= 6 | \| Treatment F3, 57= 10.63; P < 0.0001  Genotype F1, 57 = 0.4529; P = 0.5037  Interaction F3, 57= 3.142; P = 0.0321 \|  \| \| --- \| --- \| |
| Object location  CORT: *OCT2^+/+^* n= 11; *OCT2^-/-^* n= 13  CORT/FLUOX: *OCT2^+/+^* n= 5; *OCT2^-/-^* n= 7  CORT/FLUOX/TRP: *OCT2^+/+^* n= 4; *OCT2^-/-^* n= 4 | \| Treatment F2, 38 = 15.14; P<0.0001  Genotype F1, 38 = 1.901; P=0.1760  Interaction F2, 38= 2.568; P = 0.0899 \|  \| \| --- \| --- \| |
| Coat state  CORT/FLUOX: *OCT2^+/+^* n= 6; *OCT2^-/-^* n= 5  CORT/FLUOX/TRP: *OCT2^+/+^* n= 8; *OCT2^-/-^* n= 7 | \| Fluoxetine treatment 8,176 = 56.27; P < 0.0001  Genotype F3, 22= 1.122; P = 0.3616  Interaction F24, 176= 3.151; P < 0.0001 \|  \| \| --- \| --- \| |

**Supplementary table S~~4~~ Statistical analysis of z-score across depression-related variables after tryptophan supplementation (Figure 3E)**

Unpaired two-tailed student’s *t* tests

| **z-score** | **Significance** |
| --- | --- |
| CORT/FLUOX: *OCT2^+/+^* n= 6; *OCT2^-/-^* n= 6  CORT/FLUOX/TRP: *OCT2^+/+^* n= 8; *OCT2^-/-^* n= 5 | \| CORT/FLUOX *OCT2^+/+^* vs CORT/FLUOX *OCT2^-/-^*, p = 0.0137  CORT/FLUOX *OCT2^-/-^* vs CORT/FLUOX/TRP *OCT2^-/-^*, p = 0.0157 \| \| --- \| |

**Supplementary table S5 Statistical analysis of HPLC data after tryptophan supplementation (Figure 4)**

Two-way ANOVA (n = 5-7) followed by Sidak’s post hoc analysis of the effect of tryptophan supplementation. Main effects of tryptophan supplementation, genotype and/or genotype*tryptophan supplementation interaction

|  |
| --- |

| **Groups (Fig. 4A)** | **Significance** |
| --- | --- |
| Tryptophan/Plasma  CORT/FLUOX: *OCT2^+/+^* n= 5; *OCT2^-/-^* n= 7  CORT/FLUOX/TRP: *OCT2^+/+^* n= 6; *OCT2^-/-^* n= 5 | Supplementation F1,19 = 52.22; P < 0.0001  Genotype F1, 19 = 46.89; P < 0.0001  Interaction F1, 19 = 0.03468; P = 0.8542 |
| Kynurenin/Plasma  CORT/FLUOX: *OCT2^+/+^* n= 5; *OCT2^-/-^* n= 7  CORT/FLUOX/TRP: *OCT2^+/+^* n= 6; *OCT2^-/-^* n= 5 | Supplementation F1,19 = 38.29; P < 0.0001  Genotype F1,19 = 44.64; P < 0.0001  Interaction F1, 19 = 3.124; P = 0.0932 |

| **Groups (Fig. 4B)** | **Significance** |
| --- | --- |
| Tryptophan/striatum  CORT/FLUOX: *OCT2^+/+^* n= 6; *OCT2^-/-^* n= 7  CORT/FLUOX/TRP: *OCT2^+/+^* n= 6; *OCT2^-/-^* n= 5 | Supplementation F1,20 = 26.63; P < 0.0001  Genotype F1, 20 = 116.9; P < 0.0001  Interaction F1, 20 = 2.271; P = 0.1474 |
| Tryptophan/hippocampus  CORT/FLUOX: *OCT2^+/+^* n= 6; *OCT2^-/-^* n= 7  CORT/FLUOX/TRP: *OCT2^+/+^* n= 6; *OCT2^-/-^* n= 5 | Supplementation F1,20 = 9.245; P = 0.0065  Genotype F1,20 = 78.78; P < 0.0001  Interaction F1,20 = 4.455; P = 0.0476 |
| Tryptophan/cortex  CORT/FLUOX: *OCT2^+/+^* n= 5; *OCT2^-/-^* n= 7  CORT/FLUOX/TRP: *OCT2^+/+^* n= 6; *OCT2^-/-^* n= 5 | Supplementation F1,19 = 21.50; P = 0.0002  Genotype F1,19 = 110.1; P < 0.0001  Interaction F1,19 = 1.909; P = 0.1831 |
| 5-HT/striatum  CORT/FLUOX: *OCT2^+/+^* n= 6; *OCT2^-/-^* n= 7  CORT/FLUOX/TRP: *OCT2^+/+^* n= 6; *OCT2^-/-^* n= 5 | Supplementation F1,20 = 11.61; P = 0.0028  Genotype F1,20 = 8.757; P = 0.0078  Interaction F1,20 = 0.002546; P = 0.9603 |
| 5-HT/hippocampus  CORT/FLUOX: *OCT2^+/+^* n= 6; *OCT2^-/-^* n= 7  CORT/FLUOX/TRP: *OCT2^+/+^* n= 6; *OCT2^-/-^* n= 5 | Supplementation F1,20 = 9.335; P = 0.0062  Genotype F1,20 = 3.068; P = 0.0952  Interaction F1,20 = 1.811; P = 0.1935 |
| 5-HT/cortex  CORT/FLUOX: *OCT2^+/+^* n= 5; *OCT2^-/-^* n= 7  CORT/FLUOX/TRP: *OCT2^+/+^* n= 6; *OCT2^-/-^* n= 5 | Supplementation F1,19 = 1.608; P = 0.2200  Genotype F1,19 = 7.250; P=0.0144  Interaction F1,19 = 0.2478; P = 0.6243 |
| Kynurenine/striatum  CORT/FLUOX: *OCT2^+/+^* n= 6; *OCT2^-/-^* n= 7  CORT/FLUOX/TRP: *OCT2^+/+^* n= 6; *OCT2^-/-^* n= 5 | Supplementation F1,20 = 16.63; P = 0.0006  Genotype F1,20 = 79.33; P < 0.0001  Interaction F1,20 = 5.066; P = 0.0358 |
| Kynurenine/hippocampus  CORT/FLUOX: *OCT2^+/+^* n= 6; *OCT2^-/-^* n= 7  CORT/FLUOX/TRP: *OCT2^+/+^* n= 6; *OCT2^-/-^* n= 5 | Supplementation F1,20 = 3.134; P = 0.0919  Genotype F1,20 = 13.35; P = 0.0016  Interaction F1,20 = 1.440; P = 0.2442 |
| Kynurenine/cortex  CORT/FLUOX: *OCT2^+/+^* n= 5; *OCT2^-/-^* n= 7  CORT/FLUOX/TRP: *OCT2^+/+^* n= 6; *OCT2^-/-^* n= 5 | Supplementation F1,19 = 7.126; P = 0.0152  Genotype F1,19 = 30.96; P < 0.0001  Interaction F1,19 = 0.2656; P = 0.6122 |

**Supplementary table S6 Detailed data and statistical analysis of electrophysiological recordings of DR 5-HT neurons (Figure 4C)**

Mann-Whitney tests

| Firing rate (Hz) | CORT | CORT/FLX | CORT/FLX/TRP |
| --- | --- | --- | --- |
| *OCT2 ^+/+^* | 3.02 ± 0.21 (n= 65; 7 mice) | 0.72 ± 0.09 (n= 54; 7 mice) | 1.02 ± 0.13 (n= 34; 5 mice) |
| *OCT2 ^-/-^* | 2.34 ± 0.17 (n= 45; 5 mice) | 1.28 ± 0.19 (n= 45; 4 mice) | 1.28 ± 0.14 (n= 60; 5 mice) |

| **Groups** | **Significance** |
| --- | --- |
| CORT: *OCT2^+/+^* n= 65; *OCT2^-/-^* n= 45  CORT/FLUOX: *OCT2^+/+^* n= 54; *OCT2^-/-^* n= 45  CORT/FLUOX/TRP: *OCT2^+/+^* n= 34; *OCT2^-/-^* n= 60 | CORT *OCT2^+/+^* vs CORT/FLUOX *OCT2^+/+^*, P < 0.0001  CORT *OCT2^-/-^* vs CORT/FLUOX *OCT2^-/-^*, P < 0.0001  CORT/FLUOX *OCT2^+/+^* vs CORT/FLUOX *OCT2^-/-^*, p = 0.0361  CORT/FLUOX *OCT2^+/+^* vs CORT/FLUOX/TRP *OCT2^+/+^*, p = 0.0359  CORT/FLUOX *OCT2^-/-^* vs CORT/FLUOX/TRP *OCT2^-/-^*, p = 0.5723 |

**Supplementary table S7 Detailed data of 5-HT1A sensitivity (8-OHDPAT-induced hyperthermia; Figure 4D)**

Two-way ANOVA (n = 8-10) followed by Sidak’s post hoc tests

| CORT/VEH: *OCT2^+/+^* n= 9; *OCT2^-/-^* n= 10  CORT/FLUOX: *OCT2^+/+^* n= 10; *OCT2^-/-^* n= 8 | \| Fluoxetine treatment F1, 33 = 2.019; P = 0.1647  Genotype F1, 33= 6.460; P = 0.0159  Interaction F1, 33= 30.94; P = 0.5818 \|  \| \| --- \| --- \| |
| --- | --- | --- | --- |

**Supplementary table S8 Statistical analysis of Western blot data (Figure 5A)**

Two-way ANOVA (n = 6-9) followed by Tukey’s post hoc tests. Main effects of treatment, genotype and/or genotype*treatment interaction

| **Groups** | **Significance** |
| --- | --- |
| pERK1/2/ERK1/2 (% control)  Hippocampus  CORT: *OCT2^+/+^* n= 6; *OCT2^-/-^* n= 6  CORT/FLUOX: *OCT2^+/+^* n= 6; *OCT2^-/-^* n= 7  CORT/FLUOX/TRP: *OCT2^+/+^* n= 6; *OCT2^-/-^* n= 7 | Treatment F2, 32= 87.20; P < 0.0001  Genotype F1, 32 = 5.995; P = 0.0200  Interaction F2, 32= 0.8528; P = 0.4357 |
| pERK1/2/ERK1/2 (% control)  Cortex  CORT: *OCT2^+/+^* n= 6; *OCT2^-/-^* n= 6  CORT/FLUOX: *OCT2^+/+^* n= 7; *OCT2^-/-^* n= 7  CORT/FLUOX/TRP: *OCT2^+/+^* n= 7; *OCT2^-/-^* n= 7 | Treatment F2, 34= 78.72; P < 0.0001  Genotype F1, 34= 0.008852; P = 0.9256  Interaction F2, 34= 0.02774; P = 0.9727 |
| pThr389-P70S6K/β-actin (% control)  Hippocampus  CORT: *OCT2^+/+^* n= 7; *OCT2^-/-^* n= 9  CORT/FLUOX: *OCT2^+/+^* n= 7; *OCT2^-/-^* n= 6  CORT/FLUOX/TRP: *OCT2^+/+^* n= 7; *OCT2^-/-^* n= 7 | Treatment F2, 37= 16.05; P < 0.0001  Genotype F1, 37= 0.1084; P = 0.7439  Interaction F2, 37= 1.787; P = 0.1815 |
| pThr389-P70S6K/β-actin (% control)  Cortex  CORT: *OCT2^+/+^* n= 7; *OCT2^-/-^* n= 9  CORT/FLUOX: *OCT2^+/+^* n= 7; *OCT2^-/-^* n= 6  CORT/FLUOX/TRP: *OCT2^+/+^* n= 7; *OCT2^-/-^* n= 7 | Treatment F2, 37= 20.12; P < 0.0001  Genotype F1,37 = 0.001220; P = 0.9723  Interaction F2, 37= 0.01419; P = 0.9859 |
| pSer473-Akt/Akt (% control)  Hippocampus  CORT: *OCT2^+/+^* n= 7; *OCT2^-/-^* n= 9  CORT/FLUOX: *OCT2^+/+^* n= 7; *OCT2^-/-^* n= 6  CORT/FLUOX/TRP: *OCT2^+/+^* n= 7; *OCT2^-/-^* n= 7 | Treatment F2, 37= 12.06; P < 0.0001  Genotype F1, 37= 2.662; P = 0.1113  Interaction F2, 37= 4.595; P = 0.0165 |
| pSer473-Akt/Akt (% control)  Cortex  CORT: *OCT2^+/+^* n= 8; *OCT2^-/-^* n= 9  CORT/FLUOX: *OCT2^+/+^* n= 8; *OCT2^-/-^* n= 6  CORT/FLUOX/TRP: *OCT2^+/+^* n= 7; *OCT2^-/-^* n= 7 | Treatment F2, 39= 21.75; P < 0.0001  Genotype F1,39 = 0.07799; P = 0.7815  Interaction F2, 39= 1.896; P = 0.1637 |
| pSer9-GSK3β/GSK3β (% control)  Hippocampus  CORT: *OCT2^+/+^* n= 7; *OCT2^-/-^* n= 6  CORT/FLUOX: *OCT2^+/+^* n= 5; *OCT2^-/-^* n= 6  CORT/FLUOX/TRP: *OCT2^+/+^* n= 6; *OCT2^-/-^* n= 6 | Treatment F2, 31= 21.14; P < 0.0001  Genotype F1,31 = 1.816; P = 0.1876  Interaction F2, 31= 17.11; P < 0.0001 |
| pSer9-GSK3β/GSK3β (% control)  Cortex  CORT: *OCT2^+/+^* n= 7; *OCT2^-/-^* n= 7  CORT/FLUOX: *OCT2^+/+^* n= 7; *OCT2^-/-^* n= 7  CORT/FLUOX/TRP: *OCT2^+/+^* n= 7; *OCT2^-/-^* n= 6 | Treatment F2, 35= 22.43; P < 0.0001  Genotype F1,35 = 0.006246; P = 0.9375  Interaction F2, 35= 0.1075; P=0.8984 |

**Supplementary table S9 Statistical analysis of Western blot data (Figure 5B)**

Unpaired two-tailed student’s *t* tests (n = 5-7)

| **Groups** | **Significance** |
| --- | --- |
| pSer473-Akt/Akt (% control)  Hippocampus  CORT/FLUOX: *OCT2^-/-^* n= 6  CORT/FLUOX/TRP: *OCT2^-/-^* n= 5 | CORT/FLUOX *OCT2^-/-^*, vs CORT/FLUOX/TRP *OCT2^-/-^*, p = 0.0448 |
| pSer473-Akt/Akt (% control)  Cortex  CORT/FLUOX: *OCT2^-/-^* n= 7  CORT/FLUOX/TRP: *OCT2^-/-^* n= 5 | CORT/FLUOX *OCT2^-/-^*, vs CORT/FLUOX/TRP *OCT2^-/-^*, p = 0.0064 |
| pThr389-P70S6K/β-actin (% control)  Hippocampus  CORT/FLUOX: *OCT2^-/-^* n= 7  CORT/FLUOX/TRP: *OCT2^-/-^* n= 5 | CORT/FLUOX *OCT2^-/-^*, vs CORT/FLUOX/TRP *OCT2^-/-^*, p = 0.2512 |
| pThr389-P70S6K/β-actin (% control)  Cortex  CORT/FLUOX: *OCT2^-/-^* n= 7  CORT/FLUOX/TRP: *OCT2^-/-^* n= 5 | CORT/FLUOX *OCT2^-/-^*, vs CORT/FLUOX/TRP *OCT2^-/-^*, p = 0.5758 |
| pSer9-GSK3β/GSK3β (% control)  Hippocampus  CORT/FLUOX: *OCT2^-/-^* n= 7  CORT/FLUOX/TRP: *OCT2^-/-^* n= 5 | CORT/FLUOX *OCT2^-/-^*, vs CORT/FLUOX/TRP *OCT2^-/-^*, p = 0.9832 |
| pSer9-GSK3β/GSK3β (% control)  Cortex  CORT/FLUOX: *OCT2^-/-^* n= 7  CORT/FLUOX/TRP: *OCT2^-/-^* n= 5 | CORT/FLUOX *OCT2^-/-^*, vs CORT/FLUOX/TRP *OCT2^-/-^*, p = 0.4554 |

**SUPPLEMENTARY FIGURES**

**Supplementary figure S1. Total fluid intake during the sucrose preference test (average over 3 days) at basal state (BASAL), after corticosterone treatment (CORT), and after 3 weeks of fluoxetine plus corticosterone (CORT/FLUOX).** Two-way ANOVA analysis (n =10–14) shows a main effect of treatment (F2,66 = 3.529; P = 0.0350) but not genotype (F1,66 = 2.464; P = 0.1213). WT (*OCT2^+/+^*) and *OCT2* mutant (*OCT2^-/-^*) mice drink similar amounts of fluid after corticosterone and corticosterone plus fluoxetine treatment. Results are given as mean ± s.e.m.

**Supplementary figure S2. Locomotor activity during social interaction at basal state (BASAL), after corticosterone treatment (CORT), and after 3 weeks of fluoxetine plus corticosterone (CORT/FLUOX) in WT (*OCT2^+/+^*) and *OCT2* mutant (*OCT2^-/-^* ).** Two-way ANOVA analysis (n = 10–14) shows a main effect of treatment (F2,58 = 50.61; P < 0.0001) but not genotype (F1,58 = 0.0116; P = 0.9146). Results are given as mean ± s.e.m.

**Supplementary figure S3. Object location test at basal state (BASAL), after corticosterone treatment (CORT), and after 3 weeks of fluoxetine plus corticosterone (CORT/FLUOX) in WT (*OCT2^+/+^*) and *OCT2* mutant (*OCT2^-/-^* ).** Two-way ANOVA analysis (n = 10–14) shows a main effect of object (F1,60 = 45.55; P < 0.0001) and interaction object*genotype (F2,60 = 4.547; P = 0.0145) but not genotype (F2,60 = 0.005847; P = 0.9942) for WT mice (*OCT2^+/+^*); and interaction object*genotype (F2,60 = 7.553; P = 0.0012) but not treatment (F2,60 = 0.0001404; P = 0.999) or object (F1,60 = 1.936; P = 0.1692) for *OCT2* mutant mice (*OCT2^-/-^*). Sidak's post hoc, object A versus object C, *** P < 0.0001.

**Supplementary figure S4. Weight of WT (*OCT2^+/+^*) or *OCT2* mutant mice (*OCT2^-/-^*)** **during corticosterone and fluoxetine treatment.** Two-way ANOVA analysis (n = 10–14) show a main effect of time of treatment (F15,405 = 3.566; <0.0001) and of genotype (F1,405 = 7.046; P = 0.0083) but not time*genotype interaction (F15, 405 = 0.8053; P = 0.6720).

**Supplementary figure S5. 5-HT/tryptophan and kynurenine/tryptophan ratios in brain of WT (*OCT2^+/+^*) or *OCT2* mutant mice (*OCT2^-/-^*)** **during corticosterone and fluoxetine treatment with or without tryptophan supplementation.** Two-way ANOVA analysis (n = 10–14) show a main effect of genotype in striatum (F1,20 = 49.62; P <0.0001), hippocampus (F1,20 = 25.02; P = 0.0001) and cortex (F1,19 = 8.262; P = 0.0097), reflecting increased TPH2 activity. Kynurenine/tryptophan ratios ratios were comparable in all groups, suggesting no effect of genotype or of tryptophan supplementation on kynurenine production.

**Supplementary figure S6. Weight of WT (*OCT2^+/+^*) or *OCT2* mutant mice (*OCT2^-/-^*) during corticosterone plus fluoxetine treatment with (TRP) or without tryptophan supplementation.** Two-way ANOVA analysis (n = 5–6) shows no main effect of time in WT (F1.956,23.47 = 1.655; P = 0.2128) or group (F1.872,16.85 = 1.810; P = 0.1951) mice. Results are given as mean ± s.e.m.

**Supplementary figure S7. Kinetics of 8-OH-DPAT-induced hypothermia and temperature change in WT (*OCT2^+/+^*) or *OCT2* mutant mice (*OCT2^-/-^*).** Two-way ANOVA analysis (n = 8–10) shows a main effect of group (F2.138,70.56 = 20.86; P <0.0001), and of interaction group* time (F24,64 = 3.444; P <0.0001), but no main effect of time (F3,33 = 1.348; P = 0.2758) on percentage of basal temperature at the dose of 300 µg/kg; and a main effect of treatment (F1,33 = 6.206; P = 0.0179) but not of genotype (F1,33 = 2.046; P = 0.1620) or interaction (F1,33 = 0.718; P = 0.403) on percentage of decrease in temperature. Results are given as mean ± s.e.m.
